# Future Forecasting of COVID-19: A Supervised Learning Approach

**DOI:** 10.3390/s21103322

**Published:** 2021-05-11

**Authors:** Mujeeb Ur Rehman, Arslan Shafique, Sohail Khalid, Maha Driss, Saeed Rubaiee

**Affiliations:** 1 Department of Electrical Engineering, Riphah International University, Islamabad 46000, Pakistan; Arslan.Shafique@riphah.edu.pk (A.S.); S.Khalid@riphah.edu.pk (S.K.); 2 RIADI Laboratory, University of Manouba, Manouba 2010, Tunisia; maha.idriss@riadi.rnu.tn; 3 IS Department, College of Computer Science and Engineering, Taibah University, Medina 42353, Saudi Arabia; 4 Department of Industrial and Systems Engineering, College of Engineering, University of Jeddah, Jeddah 21589, Saudi Arabia; salrubaiee@uj.edu.sa

**Keywords:** COVID-19, random forest, statistical analysis, supervised learning, forecasting

## Abstract

A little over a year after the official announcement from the WHO, the COVID-19 pandemic has led to dramatic consequences globally. Today, millions of doses of vaccines have already been administered in several countries. However, the positive effect of these vaccines will probably be seen later than expected. In these circumstances, the rapid diagnosis of COVID-19 still remains the only way to slow the spread of this virus. However, it is difficult to predict whether a person is infected or not by COVID-19 while relying only on apparent symptoms. In this context, we propose to use machine learning (ML) algorithms in order to diagnose COVID-19 infected patients more effectively. The proposed diagnosis method takes into consideration several symptoms, such as flu symptoms, throat pain, immunity status, diarrhea, voice type, body temperature, joint pain, dry cough, vomiting, breathing problems, headache, and chest pain. Based on these symptoms that are modelled as ML features, our proposed method is able to predict the probability of contamination with the COVID-19 virus. This method is evaluated using different experimental analysis metrics such as accuracy, precision, recall, and F1-score. The obtained experimental results have shown that the proposed method can predict the presence of COVID-19 with over 97% accuracy.

## 1. Introduction

Over the last few decades, machine learning (ML) has gained a great deal of attention from researchers due to its capacity for solving complex real-world problems. ML can be applied to a range of significant research domains, including natural language processing, healthcare, business applications, intelligent robotic design, gaming, and image processing, among others. ML algorithms work on trial and error-based methodologies. In the development of ML models, initial training of the model is required, followed by a testing phase. Based on the results from the testing phase, error rate and misclassification are calculated [1]. One of the most popular purposes for which ML has been applied is forecasting [2,3,4,5]. Several ML algorithms have been used to predict future events in applications such as weather forecasting and disease diagnosis. In the latter case, various classification and regression algorithms, such as Support Vector Machine (SVM) and logistic regression have been used to detect different kinds of diseases [6,7]. A plethora of studies have been performed to assess how ML can predict diseases such as cardiovascular disease [8], heart disease [9], coronary artery disease [10], and breast cancer [11]. Aside from the aforementioned diseases, whose dangers but also diagnoses are richly documented, nowadays, COVID-19 presents a real global health crisis for humanity and a big new challenge to be undertaken. Today, scientists continue to perfect vaccines, but prevention and early diagnosis remain the most effective ways to protect people in the meantime. Symptoms of COVID-19 typically appear within 2 to 14 days of infection, depending on co-morbidity with Severe Acute Respiratory Syndrome (SARS). The World Health Organization (WHO) declared that the most common symptoms of COVID-19 include: a dry cough, fatigue, and flu-like symptoms, while severe cases show symptoms such as high fever, shortness of breath, and chest pain [12]. It is evident from [13] that if a person is infected with COVID-19, then the probability is high that the person will have chest pain and his/her voice will be hoarse [13]. Patients with co-morbidities, such as asthma, heart disease and pre-existing cardiovascular risk factors are more prone to COVID-19 infection, moreover these vulnerable patients exhibit worse and unpredictable outcomes [14]. Beyond the observations of these symptoms within this time period, a diagnosis often occurs around recent travel history. Severe symptoms of COVID-19 are still observed because: (1) the vaccine is a disease prevention strategy; (2) the available vaccines are not distributed on a basis of equitable and accessible sharing across countries around the world; (3) the number of people fully vaccinated is still considered very low today. Antipyretic drugs such as hydroxychloroquine have also been considered for symptomatic treatment. Beyond symptomatic treatment, though, precautions such as washing hands regularly, wearing a mask, 6 ft distancing, and washing hands reduce the spread COVID-19. As of 27 February 2021, there have been almost 3.7 million confirmed cases of COVID-19 around the globe, while more than a million people have died from the virus or its complications. Among the confirmed cases, approximately 955,000 persons have recovered [15]. Since hundreds of thousands are affected on a global scale daily, it is not possible to test every person manually. In this case, to supplement manual clinical procedures, learning algorithms that are capable of detecting future events could prove vitally helpful to detecting the virus quickly and with high accuracy. In addition, the problem of detecting COVID-19 may be solved with the help of image or textual data.

In [16], a technique for predicting COVID-19 using images by integrating a machine learning (ML) algorithm is presented, which researchers have shown achieved 86% accuracy. However, an algorithm based on images may be computationally complex, taking up precious time and resources when time is quite literally of the essence for COVID-19. Therefore, to avoid this kind of complexity, we have used textual data for the detection of COVID-19. For ML models, vast data sets are required for classification purposes [17]. This is worth noting because most researchers’ ML work on detecting COVID-19 has achieved around 90% average accuracy, which is not enough to predict such a deadly virus when the stakes are this high. Even a minor mistake may result in human death, and at the scale COVID-19 is affecting world populations, minor mistakes will magnify deaths to many thousands of times that number. Therefore, it is necessary to achieve the maximum possible accuracy in any ML model being used to detect COVID-19. In order to achieve the desired task, we have used different symptoms as features such as flu- and cold-like symptoms, throat pain, immunity, diarrhea, voice type, body temperature (Celsius), joint pain, dry cough, vomiting, breathing problems, headache, and chest pain. Chest pain can be distinguished from pneumonia in X-ray images. Pneumonia disease can be diagnosed as upper abdominal pain, especially if the lung inflammation is next to the diaphragm. Aortic dissection can be diagnosed with chest pain, abdominal pain, or both, depending upon where the dissection occurs. Therefore, if a person has pneumonia, there is a high probability that he/she will have chest pain. Based on these features, our model predicts whether or not the person is infected with COVID-19. The detail of the proposed work is given in Section 3.2.

The remainder of this paper is organized as follows: Following this Section 1 introduction, we provide a literature review of ML techniques that have been used to detect COVID-19. Section 3 then provides an overview of data representation and collection pertaining to this work. Section 3.2 is devoted to the work we are proposing. Finally, Section 4 and Section 5 provide the performance analysis and the conclusion of the proposed work, respectively. Abbreviations used in this work are listed below:

NB: Naive BayesKNN: K-nearest NeighbourML: Machine LearningDT: Decesion TreeTP: True PositivesTPR: True Positive RateFP: False PositiveLR: Logistic RegressionRF: Random forestTN: True NegativeFPR: False Positive RateFN: False Negative

### 1.1. Contributions of the Work

Given the present situation in early spring 2021, the detection and treatment of COVID-19 combine to form a high demand, and also highly demanding, research area. For the detection of COVID-19 in particular, our key findings and contributions in this work are as follows:

- We have developed a forecasting scheme that can predict the presence of COVID-19 accurately, this scheme can be easily implanted using a mobile application. The proposed approach uses classification learning algorithms such as Naïve Bayes (NB), Support Vector Machine (SVM), Decision Tree (DT), Random Forest (RF), and K-nearest neighbors (KNN). Contrarily to the traditional methods, we have used an ensemble-based method that gives higher accuracy.
- In this research, a K-fold analysis is also performed for the selection of a specific portion of the dataset, and as a result, the proposed model gives the highest accuracy.
- After performing the K-fold analysis, we have developed different learning models named “K-learning models”. These models are constructed for the implementation of ensemble-based learning techniques.
- The proposed model is validated using a number of metrics such as F1-score, accuracy, precision, and recall. In the context of COVID-19 detection, misclassifications might prove very costly in terms of human lives if a model inaccurately detects false positives, and thus we have validated the proposed model with the aforementioned accuracy metrics.

### 1.2. Motivations

COVID-19 was identified in 2019, and officially declared a pandemic by the WHO in March 2020. Since then, millions of people have been infected with COVID-19 and at least a million have died as the virus spread rapidly. Even as vaccines have been developed, hundreds of thousands of people are affected in employment, education, and other environments daily, mostly through close contact with other persons who carry the virus. Given its transmission methods and infection rate, COVID-19 cases are still increasing exponentially. These circumstances underline the need for the detection of COVID-19 presence as soon as possible, in order to protect those who do not yet have been infected. To achieve this purpose, we aim to design a machine-learning-based forecasting model that can accurately predict COVID-19 infections.

## 2. Literature Review

Natural language processing (NLP) and machine learning (ML) [18] use a significant amount of data in order to prepare pattern recognition, prediction, and explanation. Regardless of this potential downside, machine learning (ML) algorithms in particular can still be used to solve several types of problems, such as classification and regression problems. Classification is one of the most crucial tasks in text mining and can be performed using several algorithms [19,20,21,22]. Kumar et al. [23] performed various analyses on both supervised and unsupervised text mining-based classification algorithms. Chakraborti et al. [24] had proposed a methodology of detecting epilepsy using ML techniques and artificial neural networks (ANN). Sarwar et el. [25] used ML for the diagnosis of diabetes, achieving 98% accuracy.These techniques can be fruitful for the detection and diagnosis of COVID-19. Firm and accurate analysis and diagnosis of COVID-19 can save thousands, if not millions, of lives while also producing large amounts of data that can be used to train and produce more robust ML models for the detection of this deadly virus. For example, Bullock et al. [26] have claimed that deep learning (DL) and machine learning ML can replace manual diagnosis by creating accurate models that can detect disease in less time and with less complexity. ML diagnosis techniques can also be more cost effective, including for COVID-19. Computed tomography (CT) and X-ray images can be used to train ML models to achieve the desired tasks. For example, Wang and Wong [27] have proposed a deep neural network (DNN)-based technique to diagnose COVID-19 from chest radiography images, but this approach still leaves questions unanswered, such as individual intensity of symptoms and priority for treatment. Brinati et al. [16] addressed the vulnerabilities that exist in a reverse transcription-polymerase chain reaction (RT-PCR). To overcome the addressed shortcoming, two machine learning models for the detection of COVID-19 are proposed using hematochemical values which are drawn from 279 patients in which 177 patients resulted positive while the remaining are healthy persons. Although the shortcomings highlighted by Brinati et al. [16] are overcome by developing the machine learning model, but the accuracy achieved by the proposed model was 82% to 86%, which is not acceptable for accurate detection of disease. Asif et al. [28] proposed a machine learning model to categorize the healthy and COVID-19 patients. The aim was to detect COVID-19 automatically using machine learning techniques in which chest X-ray images are taken as a dataset which consists of 864 COVID-19 patients images, 1345 viral pneumonia and 1341 healthy person chest X-ray images. Using the methodology proposed in [28], the test accuracy achieved was 96%. A deep leering method nCOVnet was used for the fast detection of COVID-19 in [29] that uses X-rays of patients to detect the affected persons. For the automatic classification of COVID-19 and healthy patients, Kassani et al. [30] compared several deep learning-based feature extraction models. The extracted features are then used in a machine learning model to predict whether the person is affected by COVID-19 or not. This approach was quite fast because there is no preprocessing stage involved. To evaluate the performance of the proposed model, a publicly available COVID-19 dataset which includes the X-ray and computed tomography (CT) images was used and the accuracy of the proposed model was 98%. Yan et al. [31] proposed a prognostic prediction algorithm using ML models to predict the mortality risk of COVID-19 for specific patients. Likewise, Jiang et al. [32] proposed an ML model to detect COVID-19 affected persons, which offered approximately 80% accuracy. However, there has been much less work that uses textual data for COVID-19 prediction. In addition, the work that has already been proposed for COVID-19 detection is not accurate enough that it can be utilized to declare, in full confidence, whether or not a particular person is affected with COVID-19 or not. Therefore, by incorporating textual data, we are proposing a more accurate model that utilizes machine learning algorithms for the more accurate prediction of COVID-19. To further verify our work, we have also tested the proposed model on different machine learning algorithms such as linear regression, Naïve Bayes, decision tree, random forest, and support vector machine with different kernels (polynomial, linear, rbf, and sigmoid). After such extensive testing, we have chosen Three-Way Random Forest [33] because this is the best candidate, producing higher accuracy than the other machine learning algorithms mentioned above.

## 3. Material and Methods

**Figure 1:**
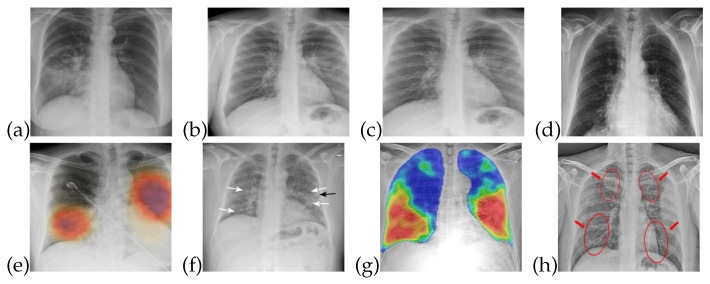
X-ray images for healthy and COVID-19 infected patients: (**a**–**d**) Normal patients X-ray (**e**–**h**) COVID-19 infected patients X-ray.

The training data for both healthy and patients infected with COVID-19 are collected from publicly accessible X-ray images [34]. The images obtained are of different sizes and image quality, so that characteristics such as contrast, sharpness, and brightness levels are different for almost all of these images. X-ray images are useful in the diagnosis of some of the rare causes of acute diarrhea. Findings on an X-ray images indicate that organic causes of acute diarrhea include intestinal dilation, irregular mucosal surface, and increased luminal fluid. For the observation of diarrhea, different patterns can be observed in X-ray images. Figure 1 shows that healthy/normal patients and COVID-19 infected patients have different patterns in their respective X-ray images. There are different patterns for recognizing the symptoms of COVID-19 that can be obtained from X-ray image. The detail of these symptoms from an X-ray images is given in [35].

When attempting to diagnose an individual with COVID-19, there are numerous necessary parameters/symptoms to consider, which include flu-like symptoms, throat pain, and immunity (gut flora), diarrhea, voice type (refers to the voice condition, i.e., voice is hoarse or normal. Value “0” is assigned for normal voice. Whereas value 1 is assigned for hoarse voice), body temp (Celsius), joint pain, dry cough, vomiting, breathing problems, chest weight, and headache. However, not all symptoms are present in every COVID-19 case, and likewise, the intensity of each symptom may differ by patient. For instance, if a person is suffering from a normal fever (98F or 99F), then it is not necessarily a given that this person has COVID-19, where fevers tend to run to more than 100 F [36]. Similarly, there are specific intensities for other COVID-19 symptoms that may be common to multiple illnesses.

### 3.1. Prepossessing

The data utilized in this work are in the form of X-ray images, which are useful for detecting lung and liver infections as well as COVID- 19 [37]. Common features that can be observed through X-ray images are chest pain, diarrhea, and viral pneumonia features [7,34,38]. In the early stages of COVID-19, though, X-ray images do not show any abnormalities related to the virus. However, as the disease progresses, abnormalities related to chest pain, diarrhea, and pneumonia do become noticeable in the X-ray images, as shown in Figure 1. In Figure 1a–d, one can see normal patient X-ray and Figure 1e–h highlights COVID-19 infected patients X-ray.

**Figure 2:**
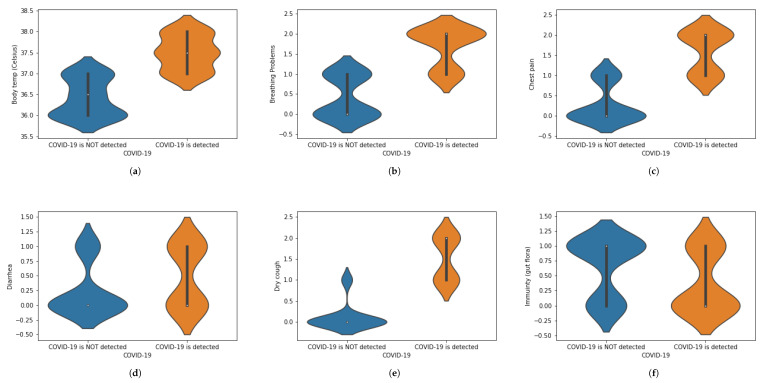

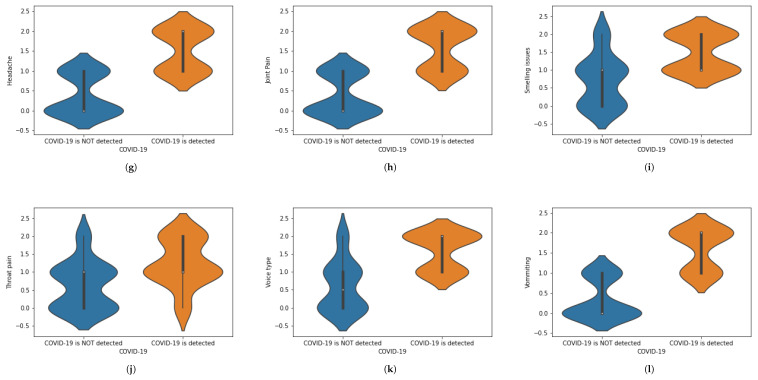
(**a**–**l**) Violin plots for corresponding to each feature used in the dataset.

We have translated this data into some statistical values. For instance, if a person is experiencing chest pain, the statistical value for the corresponding situation will be 1 and vice versa. Similarly, we have assigned numeric values corresponding to the presence of each symptom, in which 1 is assigned to ‘Yes’ and 0 is assigned to ‘No.’ When numbers other than 1 or 0 are used, these demonstrate the intensity of that symptom.The value 0 represents that a specific symptom is not present, 1 shows that the symptom is at initial stage and 2 shows that a symptom is at the highest stage. We have used general mathematical rules known as ‘Rule of Rounding. In the proposed work, we have used the data M=5000 × greater than N=13 in which *M* and *N* shows the number of rows and number of columns. Moreover, *M* shows the number of patients and *N* number of features used in the proposed work to achieve the desired task. Table 1 shows a portion of the dataset that has been used in this work. Table 1 was generated using X-ray images. These X-ray images are for healthy and COVID-19 infected patients. Features are extracted from X-ray images and different values (0, 1 and 2) are assigned. Features are extracted on the basis of the patterns observed in the X-ray images. The representation of the dataset is given in Table 2. To represent the whole dataset that is used in the proposed work, violin plots are shown in Figure 2 corresponding to each feature used in the dataset.

### 3.2. Model Selection, Training and Evaluation

The purpose of the proposed work is to classify healthy persons and COVID-19 sufferers, distinguishing them from one another. To achieve this classification, the performance of several classification learning algorithms is analyzed. The following classifiers are considered for the proposed work:

Decision Tree (DT) [39]

K-nearest neighbors (KNN) [40]

Naïve Bayes (NB) [41]

Extremely Randomized Trees (ET) [42]

Random Forest (RF) [43]

Support Vector Machine (SVM) [44]

In addition to the classifiers mentioned above, we have also considered a modified random forest algorithm, known as a three-way Random Forest (TWRF) [33]. Technically, RF is an ensemble machine learning algorithm based on the combination of different Decision Trees. RF is trained on an individual independent portion of the dataset in order to create a classifier with lower bias or variance [45]. RF works as a probability scoring classifier that assigns the weightage to every possible class. The abstentions are performed based on two thresholds, such as α, β∈ [0, 1]: here, 1 denotes the positive class, meaning that the patient is infected with COVID-19, while 0 denotes the negative class, meaning that the patient is healthy and does not have COVID-19.

To achieve the desired task, the following steps are performed:

- Take a collection of data in the form of images (X-ray images). The size of various X-ray images is different, such as *A* × *B* wherein *A* and *B* represent the rows and column of pixels. Mathematically, this can be represented as:
  $${\left[a_{ij}\right]}_{AXB}\;\;\to A\;\mathrm{and}\;B\in Z$$
- *Preprocessing stage:* Extract the features from the X-ray images and convert them into 197 numeric values such as 0, 1, and 2. (The explanation for these numerals and their assignment has been given above in Table 2.
- Make different feature vectors(F.V) F.V = f_1_, f_1_, f_2_, f_3_, …, f_14_ corresponding to each X-ray image.
- Save the F.Vs in the form of a single dataset, which can be represented as:
  (1) $$Dataset=\left(\begin{array}{c} F.V_{1}=f_{1},f_{2},f_{3},\ldots .,f_{14}\\ F.V_{2}=f_{1},f_{2},f_{3},\ldots .,f_{14}\\ F.V_{3}=f_{1},f_{2},f_{3},\ldots .,f_{14}\\ F.V_{4}=f_{1},f_{2},f_{3},\ldots .,f_{14}\\ \vdots \\ F.V_{n}=f_{1},f_{2},f_{3},\ldots .,f_{14} \end{array}\right)$$
- Split fourteen features are extracted by observing the patterns obtained from the X-ray images. For the healthy patients, the patterns that appear on the X-ray images are different from the patterns that appear on the X-ray images of the COVID-19 infected patients.
  The dataset is divided into two parts: one is for training purposes and the other for testing purposes. The training data are based on the healthy and COVID-19 infected patients’ dataset. Randomly, 80% of the dataset is selected for training purposes in which healthy and infected types of data are considered. The exact percentage of the data apportioned for training and testing can vary. In recent years, reverse transcription polymerase chain reaction (RT-PCR) has been used for the diagnosis of COVID-19 [46,47]. In [16], the shortcomings of Rt-PCR are addressed. To overcome the vulnerabilities that exist in RT-PCR, in the proposed work, machine learning techniques are employed for the real-time detection or the diagnosis of COVID-19.
  (2) $$\left\{\begin{array}{cc} \mathrm{if}\;\mathrm{Testing}\;\mathrm{data}\;\mathrm{samples} & T\;=\;20\\ \mathrm{Training}\;\mathrm{data}\;\mathrm{sample} & (\mathrm{Total}\;\text{F.Vs})\;-\;\left(20\right) \end{array}\right.$$
- Apply several classifiers to first training and then testing data in order to build the desired model. Here, the purpose of applying different classifiers is to choose the learning algorithm that best fits our proposed work.
- Select the best classifier, which can be handled by analyzing accuracy and other metrics such as precision, recall, and F1-score.
- After selecting the most suitable classifier, incorporate that classifier using voting techniques such as hard and soft voting. To perform the voting, we have developed different models for TWRF using K-fold analyses. The details of the voting techniques and K-fold analysis are given in the next few subsections.

### 3.3. Voting Techniques

Voting techniques are used to classify the test data in a more sophisticated manner. In this case, the voting techniques (a) hard voting and (b) soft voting are incorporated in order to classify this specific test data.

#### 3.3.1. Hard Voting

**Figure 3:**
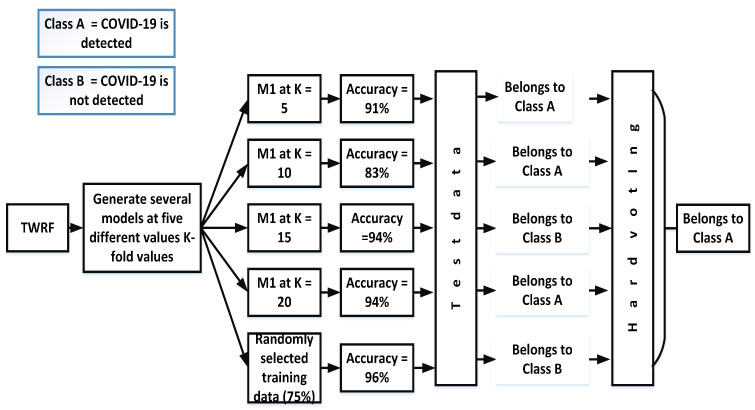
Assigning a particular class by incorporating hard voting.

Hard voting, also known as majority voting, is a technique in which priority is given to the majority. In the case of our proposed work, different models ((M_*n*_) are created by selecting different values of K-fold (K = 5, K = 10, K = 15, K = 20) and another model is also created in which training data (75%) are randomly selected. The accuracies for the different models are given in Figure 3. Based on the hard voting results, a particular class is assigned to the test data. From Figure 3, it can be seen that the votes for class *A* are three, while Class *B* has 2 votes. Therefore, the given test data belong to class *A*.

#### 3.3.2. Soft Voting

**Figure 4:**
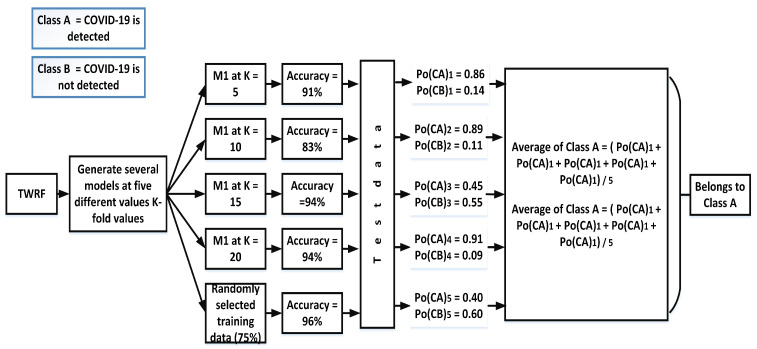
Assigning a particular class by incorporating soft voting.

In contrast to hard voting and its production of a single output, soft voting offers the possibility of either accruing or not accruing a specific class. Figure 4 shows the prediction of accruing a specific class using soft voting.

For each test, this technique gives two outputs: (a) the probability of accruing one class and (b) the probability of accruing the other class. After calculating all probabilities of both such events, an average is taken, as is demonstrated below with average of class *A* and class *B*:

$$\mathrm{For}\;\mathrm{class}\;A\;=\frac{Po{\left(CA\right)}_{1}+Po{\left(CA\right)}_{2}+Po{\left(CA\right)}_{3}+\ldots .+Po{\left(CA\right)}_{N}}{N}$$

$$\mathrm{For}\;\mathrm{class}\;B\;=\frac{Po{\left(CB\right)}_{1}+Po{\left(CB\right)}_{2}+Po{\left(CB\right)}_{3}+\ldots .+Po{\left(CB\right)}_{N}}{N}$$

For the proposed work:

$$\mathrm{For}\;\mathrm{class}\;A\;=\frac{0.96\;+\;0.96\;+\;0.97\;+\;0.98+\;0.97}{5}=97$$

$$\mathrm{For}\;\mathrm{class}\;B\;=\frac{0.86\;+\;0.89\;+\;0.45\;+\;0.91+\;0.40}{5}=0.298$$

### 3.4. K-Fold Analysis

**Figure 5:**
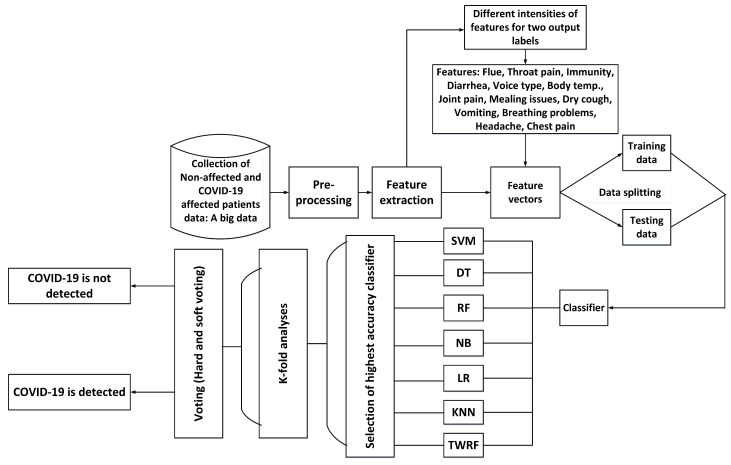
Flow diagram of the proposed work.

To properly evaluate machine learning models, K-fold validation is frequently used. Cross-validation allows the evaluation of an ML model by considering each sample of the dataset as a testing sample while the remaining are used for training purposes. Thus, in order to gauge the performance of our proposed model, we have performed 5-fold, 10-fold, 15-fold and 20-fold cross-validation tests. Figure 5 presents the flow diagram for the proposed work and its detection of COVID-19.

Apart from the K-fold validation test, we have also evaluated the proposed work in terms of accuracy, recall, precision, and F-1 score, and these results are outlined in further detail in Section 4.

## 4. Results

Here, all the steps involved in building the proposed model are implemented in Python, using pandas [48] (for pre-processing and data loading) and scikit-learn [49] (for classifier implementation). The experimentation is then executed on a system with an intel i7 processor and 12 GB RAM. The model selection and test/validation steps each required less than one second for their execution. For further evaluation of the proposed model, the following parameters are considered.

### Confusion Matrix

This is a two-dimensional array in which a number of True Positives, True Negatives, False Positives, and False Negatives prediction scores are given for any model. For our proposed work, a confusion matrix for TWRF is given in Table 3. Utilizing these results, anyone can find the accuracy, precision, and recall scores.

Table 4 shows the performance results of different machine learning algorithms for our proposed model. From Table 4, it can be seen that SVM (when the polynomial kernel is selected) and RF show comparable results. However, RF exhibits comparatively better performance than SVM. The difference between the SVM and RF performance is around 1% in terms of accuracy (SVM = 97%, RF = 98%), precision (SVM = 0.95, RF = 0.99), but in terms of recall, RF exhibits much higher values (SVM = 0.83, RF = 0.96): thus, RF is selected for the proposed work in order to make it more accurate.

For further evaluation of the proposed work, we have also calculated statistical values by selecting different instances from the dataset as testing examples. Table 5 shows the K-fold analysis for the proposed work, from which it can be seen that our model exhibits better performance at K = 10. Moreover, the average accuracy for our proposed work after applying the voting techniques is given in Table 6, which demonstrates that we achieve 97% accuracy. A performance comparison of our proposed work with several existing models is given in Table 7. The comparison is conducted based on the results given in [16,28,50,51]. However, the datasets used in the existing research work are different. The dataset we have used in our work gives better results in terms of accuracy, precision, recall and F1-score as it can be seen in Table 7.

## 5. Conclusions

The work proposed here is intended to detect the novel coronavirus, or COVID-19. This proposed model is based on machine learning algorithms that have been tested on the COVID-19 dataset of X-ray images in order to achieve the desired task. While other researchers have tried machine learning methods before, they tend to achieve only decent accuracy of around 89%-93%, but COVID-19 is a case in which it is crucial to detect a True positive. Here, False-positive events are more dangerous than False-negative, and so machine learning applications must deliver more accuracy in order to avoid false decisions and provide true efficacy as diagnostic tools. When we compared our proposed work with existing schemes, it became evident that the 97% accuracy we achieved was significantly better than most other machine learning algorithms have managed. Various evaluations of our proposed model, including testing via analysis of K-fold experimentation and comparisons of different algorithms’ performances, helped us select the most suitable machine learning algorithm to accomplish the desired task.

In future work, we will use deep learning (DL) techniques with the proposed work to increase both its efficiency and accuracy.

## Figures and Tables

**Table 1:** Some portion of the proposed dataset.

Patient No.	Flue	Throat Pain	Immunity (Gut Flora)	Diarrhea	Voice Type	Body Temp (°C)	Smelling Issues	Joint Pain	Dry Cough	Vomiting	Breathing Problem	Headache	Chest Pain	COVID-19 Yes/No
Patient-1	0	0	1	1	1	36	0	0	0	0	0	0	0	COVID-19 is not detected
Patient-2	1	0	1	0	0	36	0	0	0	0	0	0	0	COVID-19 is not detected
Patient-3	0	0	0	0	1	36	0	1	0	1	0	0	0	COVID-19 is not detected
Patient-4	1	0	0	1	0	37	1	1	0	1	0	1	0	COVID-19 is not detected
Patient-5	1	0	1	0	0	36	0	0	0	0	0	1	0	COVID-19 is not detected
Patient-6	1	0	1	0	0	37	1	1	0	0	0	0	0	COVID-19 is not detected
Patient-7	1	0	1	0	0	36.5	1	0	0	0	0	0	0	COVID-19 is not detected
Patient-8	1	0	1	0	1	37	1	1	0	1	0	0	0	COVID-19 is not detected
Patient-9	1	0	1	0	1	36	0	0	0	1	0	0	0	COVID-19 is not detected
Patient-10	1	1	1	0	1	37	1	1	0	0	1	1	0	COVID-19 is not detected
Patient-11	1	0	1	0	1	37	1	1	0	0	1	1	0	COVID-19 is not detected
Patient-12	1	0	1	0	1	37	1	1	0	0	1	0	0	COVID-19 is not detected
Patient-13	1	1	1	0	1	36	0	0	0	0	1	0	0	COVID-19 is not detected
Patient-14	1	1	1	0	1	37	1	1	0	1	1	0	0	COVID-19 is not detected
Patient-15	1	1	0	0	0	37	1	1	0	1	1	0	0	COVID-19 is not detected
Patient-16	0	1	0	0	0	36	0	0	0	0	1	1	0	COVID-19 is not detected
Patient-17	0	1	0	0	0	36	0	0	1	0	1	1	0	COVID-19 is not detected
Patient-18	0	1	0	0	0	36	0	1	0	0	1	1	0	COVID-19 is not detected
Patient-19	0	1	0	1	1	36	0	1	0	0	1	1	0	COVID-19 is not detected
Patient-20	0	1	0	1	0	36	0	1	0	0	1	1	0	COVID-19 is not detected
Patient-21	1	0	1	0	0	36	0	0	0	1	1	0	1	COVID-19 is not detected
Patient-22	1	0	1	0	0	36	0	0	0	1	0	0	0	COVID-19 is not detected
Patient-23	1	0	1	0	0	36.5	0	0	0	0	0	0	0	COVID-19 is not detected
Patient-24	1	1	0	1	1	36.5	1	1	1	0	0	1	1	COVID-19 is not detected
Patient-25	1	1	0	1	1	36.5	1	1	0	0	0	1	1	COVID-19 is not detected
Patient-26	1	1	0	1	1	36.5	1	1	0	0	0	1	0	COVID-19 is not detected
Patient-27	1	1	1	0	1	37	2	1	0	0	0	0	0	COVID-19 is not detected
Patient-28	0	0	1	0	0	36	2	0	0	0	0	0	0	COVID-19 is not detected
Patient-29	0	1	1	0	0	36.5	2	0	0	0	0	0	0	COVID-19 is not detected
Patient-30	0	1	1	0	1	37	2	0	0	0	0	0	0	COVID-19 is not detected
Patient-31	0	1	1	0	0	36	2	0	0	0	0	0	0	COVID-19 is detected
Patient-32	0	1	1	0	1	36	1	0	0	0	1	0	1	COVID-19 is detected
Patient-33	0	0	1	0	0	36	1	0	0	0	1	0	0	COVID-19 is detected
Patient-34	1	0	1	0	1	36	1	0	0	0	1	0	0	COVID-19 is detected
Patient-35	1	0	1	0	0	36.5	1	0	0	0	1	0	1	COVID-19 is detected
Patient-36	1	0	1	0	2	37	2	0	0	0	1	1	0	COVID-19 is detected
Patient-37	1	1	1	0	0	36	1	1	0	0	0	1	1	COVID-19 is detected
Patient-38	1	0	1	0	1	37	2	1	0	1	0	0	0	COVID-19 is detected
Patient-39	1	1	1	0	0	37	1	1	0	1	0	1	1	COVID-19 is detected
Patient-40	1	2	1	0	2	36	0	0	0	1	0	1	0	COVID-19 is detected
Patient-41	2	2	1	1	2	36.5	0	0	0	1	1	1	1	COVID-19 is detected
Patient-42	2	0	0	1	2	36.5	0	0	0	0	0	0	0	COVID-19 is detected
Patient-43	2	1	0	0	0	36.5	0	0	1	1	1	0	1	COVID-19 is detected
Patient-44	2	0	0	1	1	37	0	0	1	1	0	0	0	COVID-19 is detected
Patient-45	1	2	0	0	1	37	0	0	1	0	0	0	1	COVID-19 is detected
Patient-46	1	1	1	1	2	37	1	0	1	1	0	1	1	COVID-19 is detected
Patient-47	1	2	1	0	2	36	0	0	0	0	1	0	1	COVID-19 is detected
Patient-48	0	2	1	1	0	36	1	0	0	1	1	1	1	COVID-19 is detected
Patient-49	2	0	1	0	0	36	1	1	0	1	0	0	1	COVID-19 is detected
Patient-50	1	0	0	1	0	36	1	0	0	0	0	1	0	COVID-19 is detected
Patient-51	1	2	0	1	2	37	1	1	2	1	2	1	2	COVID-19 is detected
Patient-52	1	1	0	1	1	37	2	2	1	1	1	1	1	COVID-19 is detected
Patient-53	1	1	0	0	2	37.5	1	2	1	1	2	2	2	COVID-19 is detected
Patient-54	1	1	0	1	2	37.5	2	2	1	1	1	2	2	COVID-19 is detected
Patient-55	1	1	0	0	2	37.5	2	1	1	1	1	1	1	COVID-19 is detected
Patient-56	1	1	0	1	2	375	2	2	2	2	2	1	2	COVID-19 is detected
Patient-57	1	1	0	0	2	37.5	1	2	2	2	2	2	2	COVID-19 is detected
Patient-58	1	1	0	0	2	37.5	1	1	2	2	2	2	2	COVID-19 is detected
Patient-59	1	1	0	0	2	38	2	2	1	2	2	1	2	COVID-19 is detected
Patient-60	1	1	0	0	2	38	2	1	2	2	1	2	2	COVID-19 is detected

**Table 2:** Representation of dataset.

Value Assigned	Flue	Throat Pain	Immunity (Gut Flora)	Diarrhea	Voice Type	Smelling Issues	Joint Pain		Dry Cough	Vomiting	Breathing Problems	Headache	Chest Pain
Yes	1	1	1	1	1	1	1		1	1	1	1	1
No	0	0	0	0	0	0	0		0	0	0	0	0
More intense symptom	2	2	N/A	N/A	2	2	2		2	2	2	2	2

**Table 3:** Confusion matrix when test samples are 20% of the total dataset.

Total No. of Test Samples (N)	Predicted COVID-19	Predicted not COVID-19
Actual COVID-19	21	1
Actual not COVID-19	0	21

**Table 4:** Performance analysis of different machine learning algorithms for the proposed model.

Parameters	TWRF	LR	DT	RF	NB	SVM (Sigmoid Kernel)	SVM (Linear Kernel)	SVM (rbf Kernel)	SVM (Polynomial Kernel)
Accuracy	97.6	50	92	88	12	50	96	96	98
Precision	1.0	0.32	0.91	0.97	1.00	0.32	0.32	1.00	0.98
Recall	0.95	0.95	0.90	0.96	0.78	0.15	0.87	0.90	0.85
F1-score	0.97	0.47	0.90	0.96	0.87	0.20	0.46	0.94	0.91

**Table 5:** K-fold analysis.

Parameters	TWRF	LR	DT	RF	NB	SVM (Sigmoid Kernel)	SVM (Linear Kernel)	SVM (rbf Kernel)	SVM (Polynomial Kernel)
					**Accuracy analysis**				
K = 5	96	50	92	92	88	12	50	90	91
K = 10	98	50	84	85	79	21	55	84	91
K = 15	97	50	94	95	89	12	50	94	95
K = 20	95	50	95	96	90	12	50	95	95
Avg	96.5	50	91	92	87	14	51	91	93
					**Precision analysis**				
K = 5	0.95	0.32	0.91	0.92	1.00	0.32	0.32	1.00	1.00
K = 10	0.96	0.34	0.83	0.83	1.00	0.31	0.34	1.00	1.00
K = 15	0.98	0.33	0.94	0.95	0.99	0.32	0.34	0.99	0.99
K = 20	0.97	0.33	0.96	0.96	1.00	0.33	0.33	1.00	1.00
Avg	0.96	0.33	0.91	0.95	0.99	0.32	0.33	0.99	0.99
					**Rec‘ll analysis**				
K = 5	0.99	1.00	0.90	0.90	0.78	0.15	1.00	0.90	0.91
K = 10	0.98	1.00	0.80	0.80	0.60	0.32	1.00	0.79	0.92
K = 15	1.00	1.00	0.93	0.93	0.79	0.15	1.00	0.92	0.93
K = 20	0.99	1.00	0.95	0.95	0.81	0.14	1.00	0.93	0.95
Avg	0.99	1.00	0.89	0.89	0.74	0.19	1.00	0.88	0.92
					**F1-score analysis**				
K = 5	0.96	0.48	0.90	0.90	0.87	0.20	0.48	0.94	0.95
K = 10	0.96	0.50	0.81	0.81	0.75	0.31	0.50	0.88	0.95
K = 15	0.98	0.49	0.93	0.93	0.89	0.20	0.50	0.95	0.92
K = 20	0.97	0.49	0.95	0.95	0.85	0.19	0.49	0.96	0.97
Avg	96.75	0.49	0.89	0.90	0.84	0.22	0.49	0.93	0.95

**Table 6:** Average accuracy after the implementation of soft voting.

Proposed Models	When K = 5	When K = 10	When K = 15	When K = 20	Average
Accuracy	96	96	97	98	97

**Table 7:** Performance comparison of the proposed work with the existing ones.

Schemes	TWRF	LR	DT	RF	NB	SVM (Sigmoid Kernel)	SVM (Linear Kernel)	SVM (rbf Kernel)	SVM (Polynomial Kernel)
					**Accuracy analysis**				
Proposed	98	50	92	97	88	12	50	96	96
Ref [16]	78	79	81	82	81	80	79	80	81
Ref [50]	90	91	90	93	89	92	91	90	92
Ref [28]	76	75	73	77	79	78	80	81	83
Ref [51]	82	83	86	89	90	89	88	90	95
					**Precision analysis**				
Proposed	0.86	0.32	0.91	0.97	1.00	0.32	0.32	1.00	0.98
Ref [16]	0.84	0.90	0.88	0.85	0.86	0.87	0.89	0.90	0.87
Ref [50]	0.92	0.95	0.91	0.97	0.98	0.99	0.97	0.99	0.99
Ref [28]	0.97	0.98	0.96	0.99	0.98	0.99	0.98	0.99	0.98
Ref [51]	0.89	0.88	0.87	0.84	0.90	0.97	0.98	0.97	0.98
					**Recall analysis**				
Proposed	0.94	0.95	0.90	0.96	0.78	0.15	0.87	0.90	0.85
Ref [16]	0.89	0.90	0.91	0.90	0.89	0.94	0.95	0.94	0.92
Ref [50]	0.90	0.94	0.89	0.88	096	0.94	0.97	0.92	0.91
Ref [28]	0.97	0.89	0.90	0.89	0.90	0.97	0.96	0.98	0.96
Ref [51]	0.89	0.90	0.94	0.97	0.98	0.94	0.90	0.91	0.89
					**F1-score analysis**				
Proposed	0.89	0.47	0.90	0.96	0.87	0.20	0.46	0.94	0.91
Ref [16]	0.86	0.92	0.89	0.88	0.83	0.97	0.91	0.94	0.90
Ref [50]	0.90	0.92	0.89	0.90	0.91	0.89	0.98	0.96	0.94
Ref [28]	0.97	0.95	0.92	0.91	0.89	0.91	0.93	0.99	0.91
Ref [51]	0.89	0.88	0.90	0.94	0.98	0.91	0.96	0.98	0.99

## Data Availability

Public database of COVID-19 cases with chest X-ray or CT images is availabe on https://github.com/ieee8023/covid-chestxray-dataset, accessed on 11 May 2021.
